# Brain Structures Associated With Individual Differences in Somatic Symptoms and Emotional Distress in a Healthy Sample

**DOI:** 10.3389/fnhum.2020.492990

**Published:** 2020-11-17

**Authors:** Dongtao Wei, Yu Liu, Kaixiang Zhuang, Jieyu Lv, Jie Meng, Jiangzhou Sun, Qunlin Chen, Wenjing Yang, Jiang Qiu

**Affiliations:** ^1^Key Laboratory of Cognition and Personality (SWU), Faculty of Psychology, Ministry of Education, Chongqing, China; ^2^Department of Psychology, Southwest University, Chongqing, China; ^3^Department of Psychology, Central University of Finance and Economics, Beijing, China

**Keywords:** stress, psychosomatic, voxel-based morphometry (VBM), cortical-limbic system, individual differences

## Abstract

Stress-related psychosomatic responses are viewed as important risks to our physical health. Growing evidence from structural imaging studies has implicated that stress and trauma exposures have negative effects on brain structural alterations. However, whether stress-related emotional distress and somatic symptoms are related to the structure of brain systems remains unclear. Also, stress-related somatic symptoms have adverse effects on emotional distress. In turn, emotional distress may influence somatic symptom reports *via* negative cognitive bias. However, whether this relationship is mediated by specific brain morphology remains poorly understood. First, we used voxel-based morphometric approaches to investigate the neuroanatomical basis underlying somatic symptoms and emotional distress in a large sample of healthy subjects (ages 18–27 years). We found that relatively high stress-related somatic symptoms were associated with reduced gray matter volumes (GMVs) in the ventral medial prefrontal cortex (vmPFC), anterior insula, somatosensory cortex, hippocampus, and amygdala. Furthermore, a moderator analysis was performed to investigate the impact of recent stressful life events (moderators) on the association between specific GMVs (independent variables) and emotional distress (dependent variables). Interestingly, high levels of emotional distress were associated with small volumes of the vmPFC, anterior insula, hippocampus, and amygdala in participants with experience with more recent stressful life events. Finally, we performed mediation analyses to investigate the specific brain areas that mediate the association between emotional distress and somatic symptoms. The results showed that the effect of emotional distress on somatic symptoms is mediated by reductions in the volume of the hippocampus, the impact of somatic symptoms on emotional distress is mediated by the volume of the vmPFC. These results provided evidence that higher stress-related somatic symptoms are associated with smaller volume in prefrontal, insula, and limbic regions involved in emotion, interoception, and memory processing. The vmPFC and hippocampus play different roles in the relationship between emotional distress and somatic symptoms.

## Introduction

The brain is the key organ involved in stress processes, as it determines what individuals will experience as stressful and how individuals will cope with stressful experiences (McEwen and Gianaros, 2011). Acute and chronic stresses have a demonstrated influence on physical health and may trigger different types of somatic symptoms and cause the phenomena of cardiovascular issues (Steptoe and Kivimaki, 2012), pain, and insomnia (Cohen et al., 2007; Chrousos, 2009). Also, stress plays an important role in the development of somatic disorders, such as chronic fatigue syndrome, fibromyalgia, and irritable bowel syndrome (Dailey et al., 1990; O’Mahony et al., 2009; Tak and Rosmalen, 2010). Stress has also been linked to emotional distress and mental diseases, such as anxiety, depression, and post-traumatic stress disorder (Shalev et al., 1998). Previous studies have indicated that changes in brain morphology are associated with somatic symptom disorders (Davis et al., 2008; Valet et al., 2009). Moreover, one study showed that the allostatic-interoceptive brain system is not only associated with regulating peripheral system in the body but also with a wide range of psychological phenomena (Kleckner et al., 2017). The brain appraisal systems are related to both psychological stress and physiological stress reactions in the body (Gianaros and Wager, 2015). However, how individual differences in stress-related somatic symptoms and emotional distress are associated with the structure of brain systems remains unclear in nonclinical populations.

Emotional distress plays a role in the perception and somatic symptoms (Edwards et al., 2011; Perez et al., 2015). For example, depression, anxiety, and catastrophizing can frequently influence somatic symptoms or the amplification of physical sensations (Edwards et al., 2011). The cognitive-behavioral model identified that greater catastrophizing was associated with more somatic complaints in somatization disorder (Deary et al., 2007). Furthermore, a neuroimaging meta-analysis indicated that chronic pain patients demonstrated reductions not only in the matrix of regions involved in pain perception but also in other regions involved in the cognitive, affective, and perceptual domains (Smallwood et al., 2013). For example, patients with irritable bowel syndrome (ages 28–68 years) demonstrated cortical thinning in the right anterior cingulate cortex (ACC) and the bilateral anterior insula area, which are involved in the pain, attention, and homeostatic systems (Davis et al., 2008). Also, previous studies revealed that the anticipation of pain may modulate somatic sensation (pain) through hippocampal amplification (Ziv et al., 2010; Gondo et al., 2012). For instance, hippocampal activity was found to be negatively correlated with daily physical complaints mediated by the different levels of anxiety in adult healthy subjects (Gondo et al., 2012) and anticipation ratings (Ziv et al., 2010).

Also, somatic disorders are highly comorbid with anxiety and depression (Thieme et al., 2004), which may support a strong bidirectional link between emotional distress and the somatic symptoms of psychosomatic disorders. Previous studies showed that patients with mood disorders showed abnormalities in the morphology of cortico-limbic areas, such as the ACC, ventral medial prefrontal cortex (vmPFC), amygdala, insula, and hippocampus, which are involved in self-referential and emotional responses, autonomic regulation and emotional memory (Duman and Monteggia, 2006; Drevets et al., 2008; Adam Samuels et al., 2015; Schmaal et al., 2017). Previous studies have indicated that the vmPFC plays a role in the generation of emotional distress based on the perception of physiological changes in the body (Wager et al., 2009a,b; Thayer et al., 2012; Gianaros and Wager, 2015). For example, stressor-evoked vmPFC deactivation was linked to heart rate reactions and self-reported anxiety (Wager et al., 2009a,b). Therefore, whether the vmPFC volume mediates the association between emotional distress and somatic symptoms in a healthy sample is unknown.

Previous researchers suggested that the prefrontal-limbic brain circuit is known to mediate the allostatic load processes involved in experiencing and coping with stressful experiences (McEwen and Gianaros, 2010). It was also proven that the temporal dynamics of limbic-striatal and prefrontal cortical activity is related to adapting to reduce and respond to acute stress (Sinha et al., 2016). Moreover, some studies with non-psychiatric samples have indicated that decreased volume in prefrontal-limbic regions, such as the medial prefrontal cortex, anterior cingulate cortex, hippocampus, amygdala, and insula, is associated with more cumulative stressful life events (Ansell et al., 2012) and greater perceived stress (Gianaros et al., 2007). Stressful life events are also associated with subsequent increases in symptoms of depression and anxiety. Thus, the relationship between stress-related emotional distress and cortico-limbic volume may be moderated by recent stressful life events.

In this study, we first aimed to address this question by investigating whether stress-related somatic symptom and emotional distress have similar neuroanatomical mechanisms in a large sample of healthy young people. Second, moderation analyses were employed to understand the variables that affect the direction or strength of the relationship between the dependent and independent variables (Baron and Kenny, 1986). Using this analytical technique, we were able to test whether the relationship between stress-related emotional distress and local gray matter volume (GMV) is moderated by recent adverse life events. Finally, mediator analyses were employed to understand a known relationship by exploring the underlying mechanism or process by which an independent variable influences a dependent variable through a mediator variable (Baron and Kenny, 1986). Using this analytical technique, we were able to test the following: (1) whether the impact of emotional distress on somatic symptoms is mediated through the volume of pain-related areas and the hippocampus; and (2) whether somatic symptoms influence emotional distress through the volume of the vmPFC.

## Materials and Methods

### Subjects

The sample was part of our Southwest University Longitudinal Imaging Multimodal (SLIM) data, which are available for research through the International Data-sharing Initiative (INDI^1^). The goal of the project was to investigate the associations among individual differences in brain structure and function, creativity, and mental health (Wei et al., 2014; Chen et al., 2016; Tian et al., 2016; Liu et al., 2017). For detailed descriptions of the SLIM data, please refer to our recent article (Liu et al., 2017). In the present research, 329 participants (mean age: 20.42 ± 1.61 years; females = 180) were part of the SLIM data and were recruited from Southwest University by way of flyers, online advertisements, and face-to-face communication. The young adults were screened as eligible for the SLIM study if they were freshman or sophomores and were fluent in Chinese. The exclusion criteria included the following: (1) MRI-related exclusion criteria, which included claustrophobia, metallic implants, Meniere’s Syndrome, and a history of fainting within the previous 6 months; (2) current psychiatric or neurological disorders; (3) use of psychiatric drugs within the 3 months before scanning; (4) pregnancy; (5) a history of head trauma; and (6) three subjects have IQ scores below 80 were excluded. Each subject was paid for his/her participation (approximately 25–30 dollars for each MRI scan and 10 dollars for each 2-h behavioral test). All students had passed their physical examinations during their freshman year; thus, we did not use standard physical examinations. We only employed a self-report questionnaire to access their physical health. No subjects in this study had a serious physical illness during their scanning. To assess the potential mental disorders, two well-trained and experienced graduate students in the school of psychology performed the Structured Clinical Interview for the DSM-IV. The students did not meet the DSM-IV criteria for psychiatric disorders and did not use drugs that could affect brain function (including antidepressant drugs). None of them developed a psychiatric illness between the different scans. This study was approved by the Research Ethics Committee of the Brain Imaging Center of Southwest University. Informed written consent was obtained from each subject. This study was conducted following the Declaration of Helsinki, revised in 1989.

### Assessments of Psychological Variables

Recent stressful life events were assessed with the Chinese version of the Adolescent Self-Rating Life Events Checklist (ASLEC; Liu et al., 1997). The questionnaire consists of 26 items that represent several stress domains (family, school, interpersonal, individual, and so on) that evaluate the impact of stressful life events experienced within the prior year. For each event that occurred, participants have to report about the impact the event had on their lives on a 5-point Likert scale, with a response pattern ranging from 1 (“not at all”) to 5 (“extremely severe”). Scores were set to 0 for events that volunteers report did not occur in the prior year. According to the suggestions by Nikolova et al. (2012), we created a cumulative score by summing the total number of experience stressors. Cronbach’s alpha coefficient for internal consistency in this sample was 0.85, and the Spearman–Brown Split-Half coefficient was 0.74. This finding was reported in our previous study (Qiao et al., 2013).

We measured stress-related somatic symptoms and emotional distress using the Psychosomatic Tension Relaxation Inventory (PSTRI; McGuigan et al., 1980). The PSTRI focuses on the dysfunctional, negative, or less desirable response to stress, to some extent, exhibit unhealthy physiological, psychological, and behavioral responses to stress. This inventory contains 50 items describing stress-related somatic symptoms and emotional distress that participants are required to complete within 15 min *via* subjective self-report methods. Each item uses a 5-point scale from “never happens” to “always.” The inventory consists of 30 somatic complaints and 17 emotional distress items related to stress. The rest of the items are closely related to behavioral responses. The somatic symptoms included backache, stiff neck/shoulder, epigastric discomfort (including appetite loss), headache, dizziness, tachycardia/dyspnoea, weight loss, and fatigue. The emotional distress included difficulty concentrating, worrying, irritability, an overcrowded mind, loneliness, loss of interest, and health concern. The behavioral responses included drinking, smoking, and substance abuse. We created a cumulative score by summing up the total number of somatic symptoms and emotional distress scores, respectively. After a 10 weeks interval, the reliability coefficient of the PSTRI is 0.77 (McGuigan et al., 2012). The reliability of the PSTRI seems to have reached an acceptable level.

### MRI Data Acquisition

The imaging data were collected using an eight-channel head coil on a Siemens 3.0-T Siemens Trio MRI scanner (Siemens Medical Systems, Erlangen, Germany) at the Brain Imaging Center, Southwest University. A magnetization-prepared rapid gradient echo (MPRAGE) sequence was used to acquire high-resolution T1-weighted anatomical images (repetition time = 1,900 ms, echo time = 2.52 ms, inversion time = 900 ms, flip angle = 9 degrees, resolution matrix = 256 × 256 mm^2^, slices = 176, thickness = 1.0 mm, and voxel size = 1 × 1 × 1 mm^3^).

### Preprocessing of Structural Data

The MR images were processed using the SPM8 program (Wellcome Department of Cognitive Neurology, London, UK^2^) implemented in MATLAB 7.8 (MathWorks Inc., Natick, MA, USA). Each MR image was first displayed in SPM8 to screen for artifacts or gross anatomical abnormalities. For better registration, the reorientation of the images was manually set to the anterior commissure. An optimized VBM protocol was used, applying the Diffeomorphic Anatomical Registration through Exponentiated Lie Algebra (DARTEL) algorithm (Ashburner, 2007). The images were segmented into different tissue classes [gray matter (GM), white matter (WM), and cerebrospinal fluid (CSF)] and were successfully passed by visually checking for major artifacts. Subsequently, the GM imaging maps obtained by the aforementioned procedure were transformed into a GM template representing the stereotactic standardized Montreal Neurological Institute (MNI) space at a voxel size of 1.5 × 1.5 × 1.5 mm. Based on the deformation fields calculated during segmentation, a template was generated by the DARTEL algorithm. The DARTEL registration involves computing the specific template first by using the average tissue probability maps from all the participants and then warping each participant’s segmented maps to a specific template. To improve the alignment and to achieve a more accurate inter-subject registration, the procedure was repetitively conducted until the best study-specific template was generated. To ensure that regional differences in the absolute amount of GM were conserved, the image intensity of each voxel was modulated by the Jacobian determinants. The modulated images constituted the GMV. Finally, the normalized modulated images (GM maps) were smoothed with a 10-mm full-width at half-maximum Gaussian kernel to increase the signal-to-noise ratio.

### Statistical Analysis

Statistical analyses of the behavioral data were performed using the statistical software package SPSS 20 (IBM SPSS Statistics for Windows, Version 20.0, IBM Corporation, Armonk, NY, USA). To characterize the relationship between somatic symptoms, emotional distress, and recent stressful life events, we computed Pearson’s correlations between all measure pairs. Also, we used two-sample *t*-tests to examine whether there were gender differences among the measures.

Statistical analyses of the GMV data were performed using SPM8. In the whole-brain analyses, we used multiple linear regressions to identify regions where the GMV was associated with individual differences at the levels of stress-related somatic complaints and emotional distress. In the multiple linear regression analyses, the somatic complaints and emotional distress scores were used as the variables of interest. To control for possible confounding variables, age, sex, and the global GM volumes were entered as covariates into the regression model. To avoid edge effects around the borders between the GM and WM, an absolute threshold masking of 0.2 was used, meaning that voxels with GM values lower than 0.2 were excluded from the analyses. For the whole-brain analyses, the cluster-level statistical threshold was set at *p* < 0.05 (family-wise error corrected at the non-stationary cluster level) with an underlying voxel level of *p* < 0.001 (Hayasaka et al., 2004). In this non-isotropic cluster-size test of random field theory, a relatively high cluster-determining threshold combined with high smoothing values of more than six voxels leads to appropriate conservativeness in real data. With high smoothing values, an uncorrected threshold of *p* < 0.01 seems to lead to anti-conservativeness, whereas that of *p* < 0.001 seems to lead to slight conservativeness (Silver et al., 2011). Non-stationary cluster size tests can be safely applied to data known to be non-stationary (e.g., not uniformly smooth), such as VBM data (Hayasaka et al., 2004; Takeuchi et al., 2013).

In addition to the whole-brain analyses, we also performed the region-of-interest (ROI) analyses of two regions (the bilateral insula and bilateral amygdala) related to interoception (Craig, 2003, 2009), pain (Borsook et al., 2016), and anxiety (Qin et al., 2014). The bilateral insula and amygdala were defined anatomically by the automated anatomical labeling (AAL; Tzourio-Mazoyer et al., 2002) template using the WFU PickAtlas tool. The results are reported at an FWE-corrected *p* < 0.05 for a mask of the bilateral insula and amygdala on the abovementioned neuroanatomical atlas.

### Moderation and Mediation Analyses

To test the strength of the relationship between stress-related somatic complaints/emotional distress and local GMV and whether these variables were affected by recent stressful life events, we performed a moderation analysis using the interaction effect MODPROBE macro designed for SPSS and SAS (Hayes and Matthes, 2009). The *z*-scores of the recent stressful life events were entered as the moderator variables, the *z-scores* of somatic symptoms or emotional distress as focal predictor variables, and the local GMV as dependent variables in a regression analysis within SPSS Statistic-16^3^. The mean probabilistic GMV was extracted for each subject in brain regions that were significant associated with somatic symptoms. To better understand this moderation effect, we estimated and plotted different conditional effects of the focal predictor on the dependent variables at low (one SD below the moderator mean), the moderator mean, and high (one SD above the moderator mean) values of the moderator.

Besides, based on the hypothesis of a vicious circle of somatic perception and psychological factors in psychosomatic symptoms, we also performed mediation analyses to test whether the different brain regions (hippocampus and vmPFC) could explain the relationship between stress-related somatic symptoms and emotional distress. A mediating variable is a variable that is part of the causal path by which an independent variable affects a dependent variable. The mediation analyses were conducted using the indirect macro designed for SPSS (Hayes, 2017). To investigate whether the effect of emotional distress on somatic symptoms is mediating by reductions in the volume of the hippocampus, the hippocampal volume was entered as the mediator variable, the *z*-scores of the somatic symptoms were entered as the dependent variable, and the *z-scores* of the emotional distress were entered as the independent variable. Corresponding to that, to investigate the impact of somatic symptoms on emotional distress, the vmPFC was entered as the mediator variable, and the *z-scores* of the somatic symptoms were entered as an independent variable. Bootstrapped sampling was used to estimate the indirect mediation effect. In this analysis, 2,000 bootstrapped samples were drawn, and biased, corrected 95% bootstrap confidence intervals (CI) were reported. CIs that did not include zero indicated a significant indirect effect of the independent variable on the dependent variable through the mediators (Preacher and Hayes, 2008).

## Results

### Sample Descriptive Statistics

**Figure 1:**
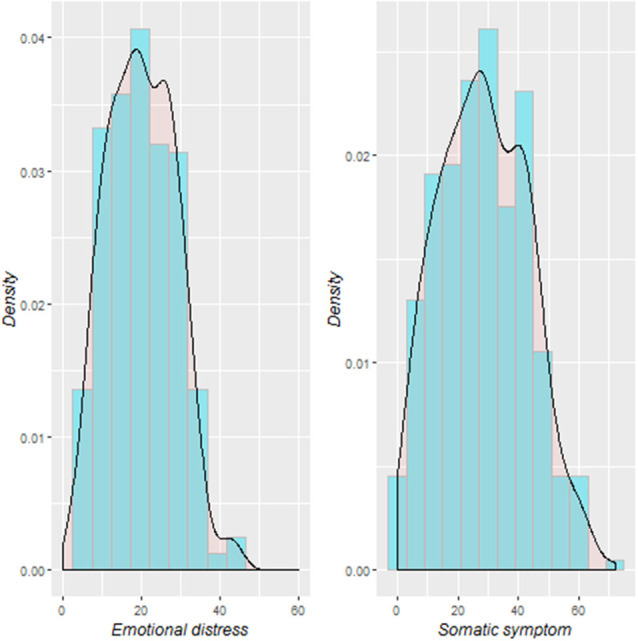
Distribution of stress-related somatic symptom and emotional distress among the study participants.

Table 1 shows a summary description of the demographic characteristics and the stress-related somatic complaints and emotional distress scores. The distribution of the stress-related somatic symptom and emotional distress scores are illustrated in Figure 1. The stress-related somatic complaint scores correlated positively with the emotional distress scores (*r* = 0.73, *p* < 0.001). The scores on the ASLEC were positively correlated the emotional distress (*r* = 0.23, *p* < 0.001) and somatic complaints (*r* = 0.25, *p* < 0.001) scores. In addition, there was a significant gender difference among the somatic complaints scores (*t* = 2.5; *p* = 0.013). The SES was not significantly associated with the stress-related somatic complaints (*r* = −0.01, *p* = 0.87) and emotional distress scores (*r* = −0.007, *p* = 0.89).

**Table 1:** Demographic and psychometric measures.

	Gender (female/male)	Range	Mean score (SD)
Participants	180/149	/	/
Age (years)	19.8 (1.3)/20.3 (1.3)	(18–27)	20 (1.3)
IQ^b^	105 (14.1)/105 (14.7)	(81–145)	105 (14.4)
Somatic complaints^a^	30.1 (14.1)/26.2 (14.7)	(0–72)	28.3 (14.5)
Emotional distress^b^	20.5 (8.2)/19 (9)	(1–45)	19.8 (8.6)
ASLEC^c^	34.2 (16.8)/37.2 (17.7)	(0–99)	35.6 (17.3)
BDI-I^b^	6.9 (5.6)/7.2 (5.7)	(0–28)	7.11 (5.65)
SES (*z*-scores)	0.13 (1.1)/–0.16 (0.91)	(–1.6–3.12)	/

*^a^There was a significant difference in the scores of somatic complaints on gender. *T* = 3.96, *p* = 0.013. A 95% confidence interval on the mean is [–7.09 –0.83]. ^b^There was no significant sex difference in emotional distress, IQ, and BDI. ^c^Adolescent self-rating life events checklist (ASLEC, Liu et al., 1997). There was no significant difference in the scores of ASLEC on gender. Note: socioeconomic status (SES) was computed as the average household income and parent’s education, where household income and education were standardized to z-scores*.

### VBM Results

**Figure 2:**
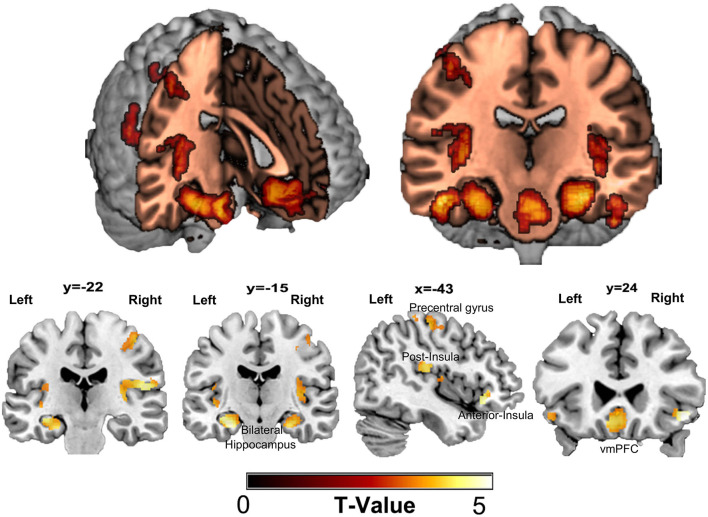
Regional gray matter volume correlated with scores of somatic symptoms. A negative correlation was found between gray matter volume (GMV) and the score of somatic symptom in some clusters that mainly included areas in the ventral medial prefrontal cortex (vmPFC), bilateral hippocampus, bilateral amygdala, bilateral somatosensory cortex, and extend into the posterior insula, inferior frontal cortex adjoin to the anterior insula. There was no significant positive correlation between GMV and the scores of psychological responses based on FWE-corrected.

A summary of the regions in which smaller GMVs were observed for the stress-related somatic symptom is provided in Table 2. There were different neuroanatomical patterns observed between the somatic symptom and emotional distress. Specifically, after controlling for possible confounding variables, including age, sex, emotional distress, and global volumes of GM, a negative correlation was found between the GMV and the somatic symptom scores in some clusters, which mainly included areas in the vmPFC, bilateral hippocampus, bilateral amygdala, bilateral somatosensory cortex and extended into the posterior insula and the inferior frontal cortex adjacent to the anterior insula as shown in Figure 2. Also, the relationship between stress-related somatic symptom and regional GMV is not influenced by depressive level, when the depressive level was also entered as a control variable in the multiple linear regression analyses. However, there was no significant negative correlation between GMV and the emotional distress scores after controlling for age, sex, somatic symptoms, and global volumes of GM. Also, there was no significant positive correlation between GMV and the somatic complaint scores or emotional distress based on the FWE-corrected results. Also, we didnot find a significant correlation between the stressful event and GMV based on the corrected results.

**Table 2:** Summary of the gray matter volume (GMV) associations with somatic symptoms.

Brain regions	MNI coordinates			Voxel size	Peak *T*-value
	*x*	*y*	*z*		
^a^Left Primary somatosensory cortex/posterior insula	−54	−19	13	649	−4.34
^a^Right Primary somatosensory cortex/posterior insula	54	−21	10	615	−3.94
^a^Ventral medial prefrontal cortex (vmPFC)	3	21	−13	3,824	−4.18
^b^Right hippocampus	30	−11	−22	458	−4.31
^b^Left hippocampus	−27	−15	−21	470	−4.43
^a^Inferior frontal cortex/anterior insula	−50	40	−19	1,100	−4.16
^b^Right amygdala	28	−8	−21	110	−4.01
^b^Left amygdala	−30	−8	−21	74	−3.96

*^a^Results are *p* < 0.05, FWE corrected for multiple comparisons at a cluster level with non-stationary correction, with an underlying voxel level of *p* < 0.001, uncorrected under whole-brain analyses. ^b^The bilateral insula and amygdala were defined anatomically by the automated anatomical labeling (AAL) using the WFU PickAtlas Tool. Results are reported at an FDR-corrected *p* < 0.05*.

Based on the hypothesis of a vicious circle of somatic perception and psychological factors in psychosomatic symptoms, we performed mediation analyses. Emotional distress was negatively associated with the volume of the hippocampus (*β* = −0.11, *p* < 0.05), and the volume of the hippocampus were significantly negatively associated with somatic symptoms (*β* = −0.14, *p* < 0.001). Meanwhile, the relationship between somatic symptom and emotional distress was modulated by the individual differences in the volume of the bilateral hippocampus. The indirect effect of emotional distress on somatic symptoms was also significant (indirect effect = 0.02, CI = [0.002 0.038]). On the other hand, emotional distress was positively associated with the volume of the vmPFC (*β* =0.08, *p* < 0.05), and the volume of the vmPFC were significantly negatively associated with somatic symptoms (*β* = −0.22, *p* < 0.001). The relationship between somatic and emotional distress was modulated by individual differences in the volume of the vmPFC, and the indirect effect of somatic symptoms on emotional distress was significant (indirect effect = −0.02, CI = [–0.041 –0.004]).

**Figure 3:**
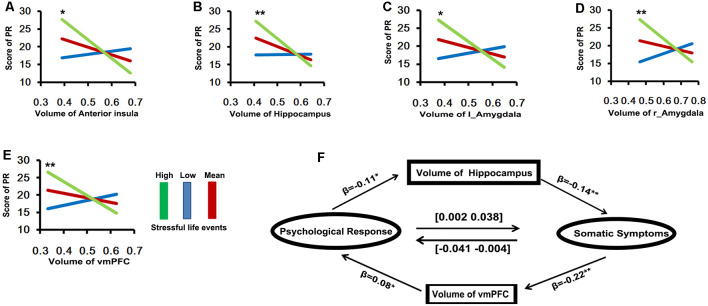
The results of moderation and mediation analyses (**A–E**, Y axis is the scores of psychological response; X axis is the volume of brain area). **(A)** The relationship between psychological response and the volume of anterior insula was moderated by recent stressful life event (*r*^2^change = 0.0284, *p* = 0.0015), such that high levels of stress-related psychological response were associated with smaller volume of anterior insula for participants with experience more recently stressful life event (green line: >1 SD above the mean, *p* = 0.0027, [–64.55 –13.62]). **(B)** The recent stressful life event also has significant moderate effect on relationship between psychological response and the volume of bilateral hippocampus (*r*^2^change = 0.0229, *p* = 0.004, green line: >1 SD above the mean, *p* = 0.0059, [–77.67 –13.21]). **(C,D)** Amygdale: (left: *r*^2^change = 0.034, *p* < 0.001, green line: >1 SD above the mean, *p* = 0.003, [–55.36 –11.29]; right: *r*^2^change = 0.029, *p* = 0.0015, green line: >1 SD above the mean, *p* = 0.0028, [–66.76 –13.97]). **(E)** VMPFC (*r*^2^change = 0.033, *p* < 0.001, green line: >1 SD above the mean, *p* = 0.005, [–58.76 –10.64]). **(F)** The relationship between somatic (X) and psychological responses (Y) was mediated by the individual difference in the volume of vmPFC (M), the indirect effect of somatic symptoms on psychological response is significant (indirect effect = −0.02, CI = [–0.041 –0.004]). Meanwhile, the relationship between somatic (Y) and psychological responses (X) was mediated by the individual difference in the volume of bilateral hippocampus (M), the indirect effect of psychological response on somatic symptoms is also significant (indirect effect = 0.02, CI = [0.002 0.038]).

To examine the strength of the relationship between local GMV and stress-related somatic symptoms/emotional distress and whether these variables were affected by recent stressful events, we performed a moderation analysis. As shown in Figure 3, the stress-related emotional distress was not significantly correlation with the local GMV. However, the relation between emotional distress and the volume of the vmPFC was moderated by recent stressful life events (*r*^2^change = 0.033, *p* < 0.001), such that high levels of stress-related emotional distress were associated with smaller volumes of the vmPFC for participants who experienced more recent stressful life events (>1 SD above the mean, *p* = 0.005, [–58.76 –10.64]). In contrast, the individuals who encountered either intermediate-level (mean, *p* = 0.437, [–25.43 11.01]) or low-level (<1 SD below the mean, *p* = 0.097, [–3.74 44.3]) stressful life events did not show significant correlations between emotional distress and the volume of the vmPFC. Likewise, recent stressful life events also had significant moderate effects on the relationship between emotional distress and the volumes of the amygdala (left: *r*^2^change = 0.034, *p* < 0.001, >1 SD above the mean, *p* = 0.003, [–55.36 –11.29]; right: *r*^2^change = 0.029, *p* = 0.0015, >1 SD above the mean, *p* = 0.0028, [–66.76 –13.97]), bilateral hippocampus (*r*^2^change = 0.0229, *p* = 0.004, >1 SD above the mean, *p* = 0.0059, [–77.67 –13.21]) and right IFG/anterior insula (*r*^2^change = 0.0284, *p* = 0.0015, >1 SD above the mean, *p* = 0.0027, [–64.55 –13.62]).

## Discussion

In the present study, we found that relatively high stress-related somatic symptoms were associated with reduced GMVs in the vmPFC, paralimbic areas, somatosensory cortex, hippocampus, and amygdala. There was no significant correlation between stress-related emotional distress and the GMV of the brain. Interestingly, the interaction between the emotional distress scores and the recent stressful events was associated with the local GMV in areas such as the vmPFC, paralimbic areas, hippocampus, and amygdala. Also, the relationship between somatic and emotional distress was mediated by individual differences in the volumes of the vmPFC and bilateral hippocampus. The results of our study provided novel evidence for the pathways of brain-psychological-physical activity (BPP) in stress-related psychosomatic symptoms.

Stress-related somatic symptoms were negatively associated with GMVs in the vmPFC, insula, and somatosensory cortex within a large sample of healthy volunteers. These findings are partially consistent with the previous results of neuroimaging studies that examined patients with somatic symptom disorders compared to healthy controls, finding that GM decreases in the medial prefrontal, cingulated, and insular cortex (Kuchinad et al., 2007; Valet et al., 2009) were involved in the processing and emotional modulation of pain. However, as their sample consisted of middle-aged and elderly patients with pain disorders, the GMV decrease in these areas may have been the consequence of long-term somatic disease, whereas our study examined individual differences in the stress-related somatic symptoms associated with differences in local GMVs in nonclinical young populations. Thus, we speculated that high levels of somatic symptom with reduced brain volume in these regions may serve as a precursor to the future development of somatic symptom disorders. Also, it is well established that individual variability in pain, visceral and thermal sensitivity is associated with GM decreases in pain-related areas and the somatosensory cortex within healthy volunteers (Erpelding et al., 2012; Elsenbruch et al., 2014). For example, previous studies used a psychophysical session to measure individual pain, visceral and thermal threshold and found that greater pain and thermal sensitivity correlated with cortical thickening in the somatosensory cortex and mid-cingulate cortex in healthy volunteers (Erpelding et al., 2012); increased visceral sensitivity also correlated with reduced GMVs in the insula, vlPFC, OFC, posterior cingulate cortex and thalamus in healthy volunteers (Elsenbruch et al., 2014). In the present study, the decreased volumes of the insula and somatosensory cortex may have been influenced by individual variability in pain and visceral sensitivity.

Greater somatic symptoms were also associated with smaller volumes in the bilateral hippocampal and amygdala regions. Reduced volumes of the hippocampus and amygdala have mainly been found in stress-related disorders, such as major depressive disorder, anxiety disorder, and post-trauma stress disorder (Weniger et al., 2009; Adam Samuels et al., 2015; Otte et al., 2016). For example, meta-analyses found that the volumes of the hippocampus and amygdala were smaller in patients with MDD than in healthy controls (Schmaal et al., 2016), this finding may have been associated with the stress-related increase in glucocorticoids, which may result in the regression of dendritic processes and loss of neurons (Sapolsky, 2000). However, these studies tended to focus on the effects of vulnerability stress, affective components, course of the disease, and age of onset on hippocampal volume reductions. Previous studies also indicated that pain-related disorder and somatic complaints were associated with decreased volumes of these regions (Gondo et al., 2012; Maleki et al., 2012; Vachon-Presseau et al., 2013). Vachon-Presseau et al. (2013) indicated that patients with chronic back pain have higher levels of cortisol than healthy controls, and higher cortisol levels were associated with smaller hippocampal volumes related to anticipatory anxiety and associative learning (Vachon-Presseau et al., 2013). Moreover, the pain-related responses in the hippocampus of pain patients have been linked to daily complaints (Gondo et al., 2012), patients with high frequencies of migraine attacks also showed smaller hippocampal volumes (Maleki et al., 2012). Together, these findings supported that decreased volumes of these regions were associated with mood disorders and pain-related somatic disorders. In our study, somatic symptoms were measured using a self-report method, which may have been influenced by individual somatosensory amplification and negative reporting bias. Nevertheless, the somatosensory and cognitive amplification contributes to the pathophysiology of somatization (Duddu et al., 2006; Perez et al., 2015). Moreover, the somatosensory amplification, and negative reporting bias may be mediated by large-scale neural systems, such as the ACC, insula, amygdala and hippocampus (Perez et al., 2015). Interestingly, high levels of emotional distress were also associated with smaller volumes of the vmPFC, anterior insula and subcortical structures in participants who experienced more recent stressful life events. Because our samples consisted of healthy subjects, we could speculate that the higher somatic symptom associated with reduced volumes of prefrontal-limbic regions may be mediated by individual differences in negative emotion and cognitive amplifiers of visceral-somatic processing.

Besides, high levels of stress-related emotional distress in individuals who experienced more recent stressful life events were also associated with smaller volumes of cortico-limbic circuits, including the vmPFC, anterior insula, hippocampus, and amygdala. Prior research has indicated that cumulative adversity over the lifetime is associated with smaller volumes in prefrontal and limbic-striatal stress-related brain regions, and reduced brain volume in these regions may partially mediate vulnerability for depression, addiction, and other stress-related psychopathologies (Ansell et al., 2012). Though we did not find a direct association between stressful life events and the GMV of the brain, we found that the interaction between more stressful life events and higher emotional distress was related to smaller volumes of the cortico-limbic circuit. The cortico-limbic circuit is known to regulate stress and emotional arousal (McEwen, 2007; McEwen and Gianaros, 2011), and decreased brain volume in key regions of this circuit may generate a risk for mental disease. In the context of the current findings, the volume reductions in the cortico-limbic regions were greatest for the individuals who experienced more stressful life events and who also reported higher stress-related emotional distress. These results suggested that physiological and emotional distress may share common neuroanatomical mechanisms in the context of individuals who experience more stressful life events.

Through the mediating effect analysis, our results further showed that the effect of emotional distress on somatic symptoms was mediated by the volume of the hippocampus. Somatic symptom reporting may be influenced by emotional reactivity through somatic sensitivity and negative reporting style (Aronson et al., 2006). Moreover, sensitization plays a role in individual variability within the tolerance and acceptance of somatic complaints (Eriksen and Ursin, 2004). It has been suggested that the hippocampus amplifies aversive events to prime behavioral responses during anxiety (Ploghaus et al., 2001; Gondo et al., 2012), for example, Ploghaus et al. (2001) demonstrated that pain-related anxiety is associated with activation changes in the hippocampal formation (Ploghaus et al., 2001). These studies suggested an amplifying role for the hippocampus in visceral-somatic processing during emotional reactivity. In contrast, the impact of somatic symptoms on emotional distress may be mediated by the volume of the vmPFC. Many human and animal studies have indicated that changes in transient stressor-evoked cardiovascular function are associated with activity in the medial prefrontal cortex (Resstel and Correa, 2006; Gianaros and Wager, 2015). The vmPFC is an important area for stress- and threat-related ANS visceromotor control and plays a role in the generation of emotional distress based on the perception of physiological changes in the body (Wager et al., 2009a,b). However, we should note that our results are based on structural imaging, thus, we did not direct test the relationship between hippocampal/vmPFC activity and the psychosomatic responses. Moreover, whether the relationship between somatic symptom and emotional distress is mediated by the volumes of the vmPFC and hippocampus needs to be further verified in a longitudinal study.

There were several limitations to the present findings. One limitation was that with the questionnaire of stressful life events, somatic symptoms and emotional distress were assessed by subjective reports, which may have led to bias or errors. Future research should consider investigating the relationship between brain structure and multiple variables (e.g., objective and subjective measurement of psychosomatic response). Second, the results of the moderation and mediation analyses must be interpreted cautiously because the effect size tended to be relatively small. Also, we used the total score of stressful life events instead of multi-dimensions. The reason for this was mainly because a cumulative score represents the total number of experienced stressors. Third, the analyses were correlational, and a longitudinal design is needed to determine the causal direction between the psychosomatic responses and the changes in brain structure in a further study. In conclusion, the college student participants in this study represented a non-psychiatric community sample; small volumes of regions of the cortical-limbic system were associated with high psychosomatic reactions and may serve to mediate vulnerability for depression, anxiety, and other stress-related psychopathologies.

## Data Availability Statement

Publicly available datasets were analyzed in this study. This data can be found here: http://fcon_1000.projects.nitrc.org/indi/retro/southwestuni_qiu_index.html.

## Ethics Statement

The studies involving human participants were reviewed and approved by Research Ethics Committee of the Brain Imaging Center of Southwest University. The patients/participants provided their written informed consent to participate in this study.

## Author Contributions

DW and JQ designed the experiments. JM, DW, KZ, QC, and WY carried out the experiments. DW and JQ analyzed the data and wrote the manuscript. YL and JL edited and revised the manuscript. All authors contributed to the article and approved the submitted version.

## Conflict of Interest

The authors declare that the research was conducted in the absence of any commercial or financial relationships that could be construed as a potential conflict of interest.

## Footnotes

^1^http://fcon_1000.projects.nitrc.org/indi/retro/southwestuni_qiu_index.html

^2^http://www.fil.ion.ucl.ac.uk/spm/software/spm8/

^3^http://www.spss.com
